# Case Report: Pemphigus following COVID-19 mRNA vaccination: two cases achieving long-term remission without relapse

**DOI:** 10.3389/fimmu.2026.1721035

**Published:** 2026-02-10

**Authors:** Yuri Fukunaga, Natsuko Saito-Sasaki, Eri Ohta, Yu Sawada

**Affiliations:** Department of Dermatology, University of Occupational and Environmental Health, Kitakyushu, Japan

**Keywords:** case study, COVID-19, pemphigus, pemphigus foliaceus, pemphigus vulgaris

## Abstract

Autoimmune blistering diseases (AIBDs) are mediated by autoantibodies targeting structural proteins of the epidermis or dermo-epidermal junction. Since the introduction of COVID-19 mRNA vaccines, isolated cases of pemphigus temporally associated with vaccination have been described, although causal relationships remain uncertain. We report two patients who developed pemphigus following COVID-19 mRNA vaccination and showed an exceptionally favorable clinical course. A 66-year-old man developed pemphigus foliaceus one week after his second vaccine dose, and a 45-year-old woman developed pemphigus vulgaris two months after her second dose. Both patients responded promptly to corticosteroid therapy, with normalization of autoantibody titers and achievement of long-term complete remission lasting over two years without relapse or maintenance therapy. These outcomes stand in contrast to sporadic pemphigus, which usually follows a chronic relapsing course, and suggest that vaccine-related pemphigus may, in rare instances, represent a transient and self-limited immune dysregulation. Such cases are exceptional and should not discourage COVID-19 vaccination.

## Introduction

The COVID-19 pandemic has led to the unprecedented deployment of mRNA vaccines worldwide (1). These vaccines elicit strong protective immunity against SARS-CoV-2 through the induction of both humoral and cellular responses (2). However, in rare cases, aberrant immune activation may occur in genetically or immunologically predisposed individuals, potentially leading to the development of autoimmune diseases (3).

Autoimmune blistering diseases (AIBDs) including pemphigus and bullous pemphigoid (BP) are mediated by autoantibodies directed against structural proteins of the epidermis or dermo-epidermal junction (4). Although AIBDs occurring in temporal association with mRNA vaccination have emerged (5), potential causal relationships remain controversial.

Herein, we describe two patients who developed pemphigus following COVID-19 mRNA vaccination and demonstrated an atypical clinical course characterized by long-term complete remission without relapse, a phenomenon that is exceptionally rare since pemphigus typically does not remit spontaneously. These cases underscore the possibility that vaccine-related pemphigus may represent a transient, self-limited immune dysregulation rather than persistent autoimmunity in some cases, and they provide reassurance regarding the overall safety of COVID-19 vaccination.

## Case reports

### Case 1

**Figure 1:**
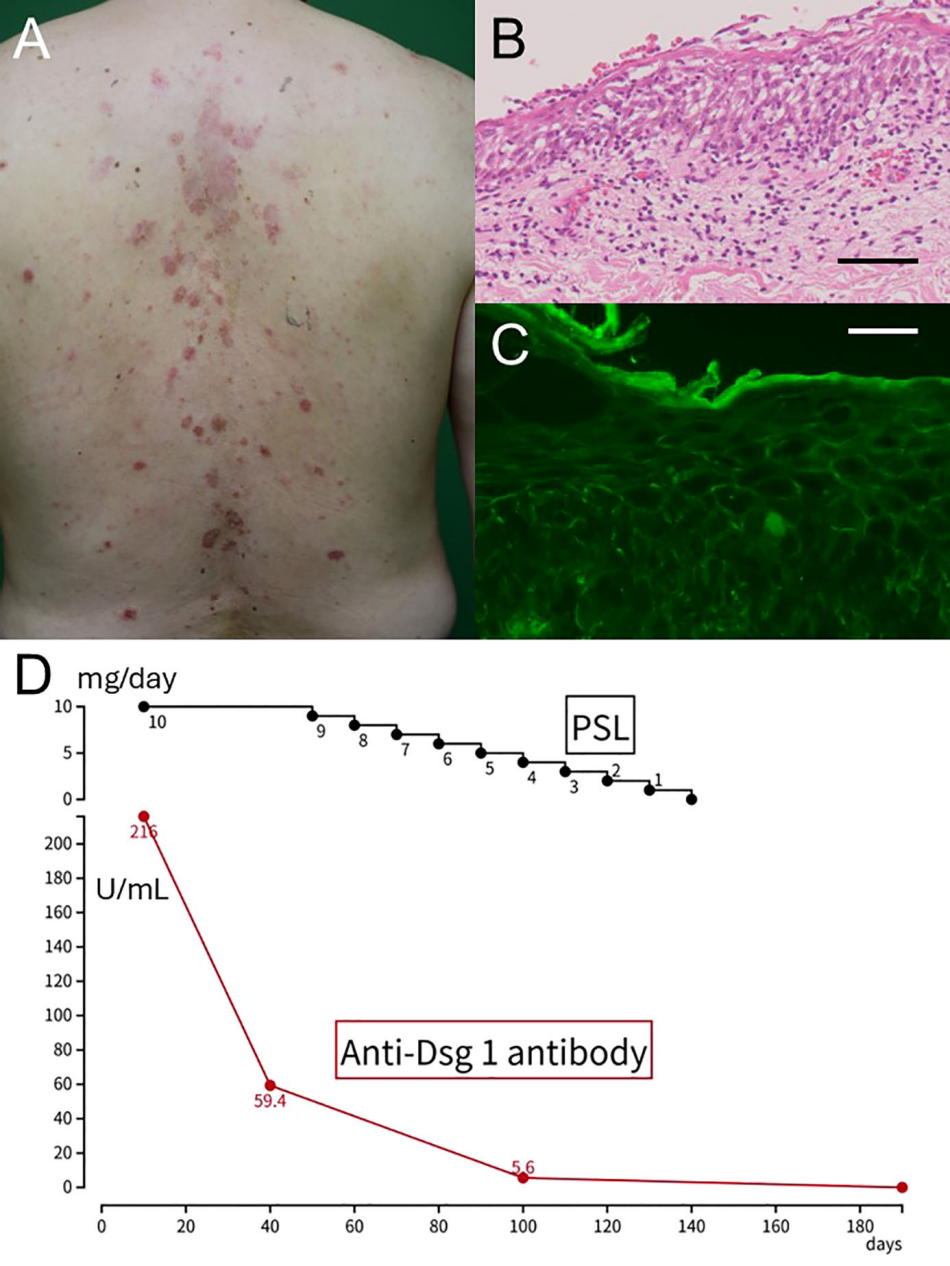
Clinical manifestation, histopathological examination, and clinical course of Case 1. **(A)** Multiple erythematous, scaly plaques on the trunk. **(B)** A skin biopsy showing spongiosis and subcorneal acantholysis with superficial perivascular lymphocytic infiltration. Scale bar: 400μm **(C)** Direct immunofluorescence demonstrating intercellular IgG deposition in the epidermis. Scale bar: 100μm **(D)** Schematic illustration summarizing the clinical course.

A 66-year-old Japanese male presented with erythematous, scaly plaques on the scalp and upper back. The eruption appeared one week after receiving his second dose of the COVID-19 mRNA vaccine (BNT162b2, Pfizer-BioNTech). He had no past medical history, took no medications or supplements, and had no previous dermatologic conditions. Neither he nor his family members had ever been infected with COVID-19 before the onset of the eruption. No potential triggers such as recent infections or new medications were identified, and his family history was negative for autoimmune blistering diseases. On physical examination, multiple circular erythematous plaques with superficial scaling were observed without mucosal involvement (**Figure 1A**). Nikolsky’s sign was negative. Laboratory findings showed a markedly elevated anti-desmoglein 1 antibody titer of 216 U/mL, while anti-desmoglein 3 was negative. Skin biopsy demonstrated spongiosis and subcorneal acantholysis in the epidermis, accompanied by superficial perivascular lymphocytic infiltration in the dermis (**Figure 1B**). DIF showed intercellular IgG deposition within the epidermis (**Figure 1C**). Based on these findings, we diagnosed his skin eruption as pemphigus foliaceus. His Pemphigus Disease Area Index (PDAI) skin activity score was 13, indicating moderate disease severity. He was initiated on oral prednisolone 10 mg/day (0.15 mg/kg), resulting in rapid clinical improvement within two weeks (**Figure 1D**). Follow-up serology demonstrated normalization of anti-desmoglein 1 titers within three months. The corticosteroid was tapered off over the following two months. He has remained in complete remission for over two years, without relapse or requirement for additional immunosuppressive therapy.

### Case 2

**Figure 2:**
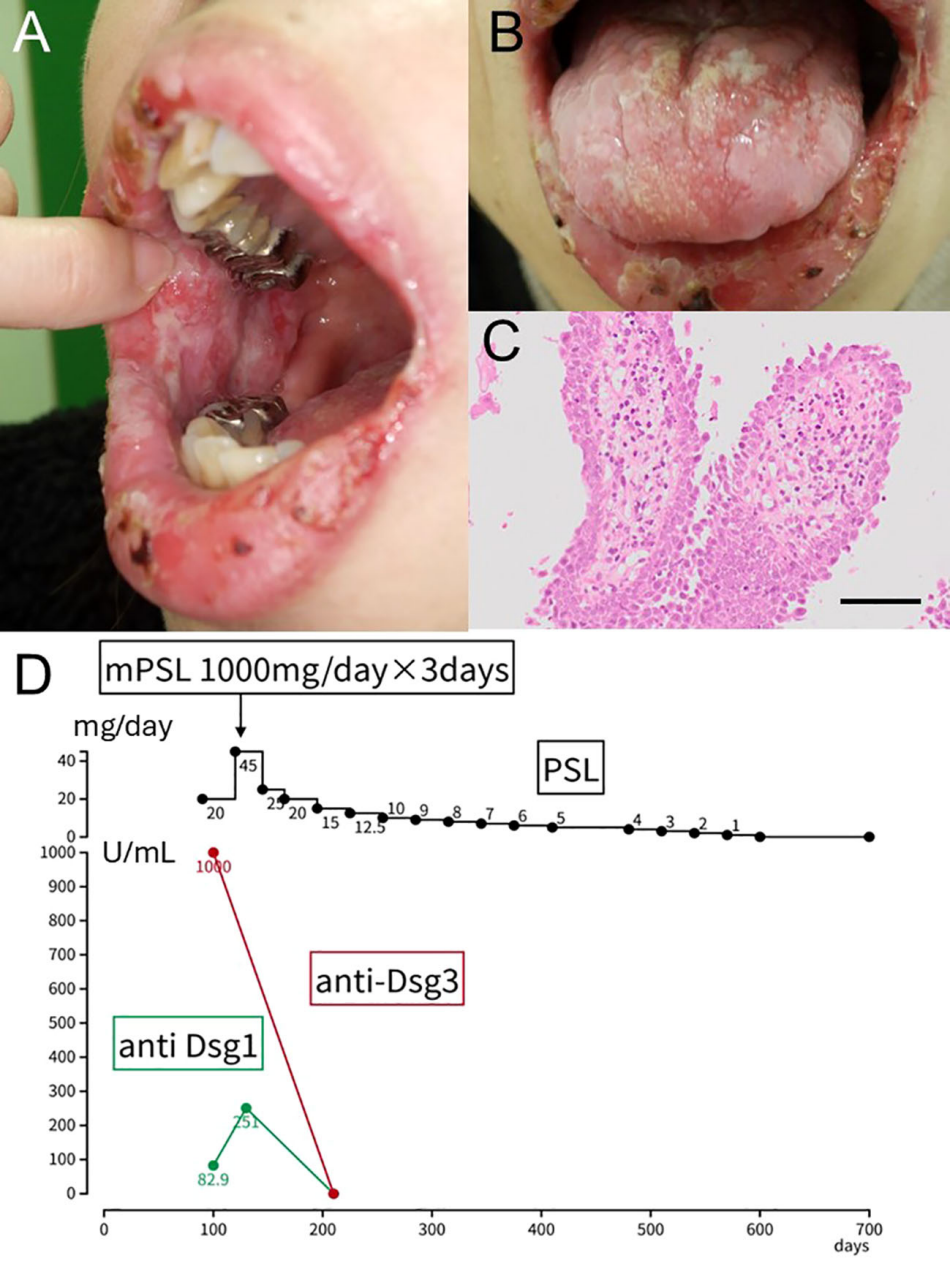
Clinical manifestation, histopathological examination, and clinical course of Case 2. **(A, B)** Multiple painful erosions on the buccal mucosa and palate. **(C)** A biopsy specimen from the oral mucosa showing suprabasal cleft formation with epithelial detachment. Scale bar: 400μm **(D)** Schematic illustration summarizing the clinical course.

1. A 45-year-old otherwise healthy Japanese female presented with progressive painful oral erosions. Symptoms developed approximately two months after receiving her second COVID-19 mRNA vaccine dose (BNT162b2, Pfizer-BioNTech). She had no comorbidities, took no medications or supplements, and had no prior dermatologic conditions. Neither she nor her family members had ever been infected with COVID-19 before the onset of her symptoms. No potential triggers such as recent infections or new medications were identified, and her family history was negative for autoimmune blistering diseases. Because the oral lesions were extremely painful, she could not consent to an additional perilesional biopsy for direct immunofluorescence, and only a single biopsy was performed. On physical examination, multiple erosions were noted on the buccal mucosa and palate (**Figures 2A, B**). She denied systemic symptoms such as fever or malaise. Serological evaluation revealed an anti-desmoglein 1 antibody level of 82.9 U/mL and an anti-desmoglein 3 antibody level >1000 U/mL, supporting the diagnosis of pemphigus vulgaris. A biopsy specimen from the oral mucosa demonstrated suprabasal cleft formation with detachment of the epithelium (**Figure 2C**). No additional biopsy was taken, and the only biopsy performed was the clinically necessary biopsy obtained as part of routine care. Her PDAI mucosal activity score was 12, consistent with moderate mucosal disease. Despite treatment with oral prednisolone 0.5 mg/kg/day (30 mg/day) for four weeks, her mucosal erosions showed minimal improvement (**Figure 2D**). Consequently, intravenous methylprednisolone pulse therapy (1 g/day for 3 consecutive days) was administered, resulting in dramatic clinical improvement within one week. Autoantibody titers became negative six months after treatment initiation. The patient has remained in complete remission for over two years without maintenance therapy. Notably, she subsequently contracted SARS-CoV-2 infection; however, she did not experience any pemphigus flare.

## Discussion

Although most cases occur sporadically, environmental triggers such as drugs, infections, and vaccinations have been implicated in the onset of pemphigus. With the global introduction of mRNA-based COVID-19 vaccines, isolated cases of autoimmune blistering diseases have been reported (6), raising questions regarding potential causal associations. The mRNA vaccines against COVID-19 exert their potent immunogenicity through both the encoded spike protein and the adjuvant-like properties of lipid nanoparticles, stimulating innate immune responses and transiently disturbing immune tolerance in predisposed individuals (7). This transient immune activation might be one of the possible pathogeneses of our cases. Another possibility is bystander activation of autoreactive lymphocytes during systemic immune stimulation, which could transiently facilitate the production of pathogenic autoantibodies (8). Molecular mimicry between SARS-CoV-2 spike protein and desmogleins has not been convincingly demonstrated, suggesting that spike-specific cross-reactivity is less likely to be responsible, and that transient nonspecific immune dysregulation is more plausible. Consistently, one patient remained free of relapse even after subsequent SARS-CoV-2 infection, suggesting that the pathogenesis in these cases may reflect transient immune dysregulation possibly due to COVID-19 vaccine. Interestingly, our two cases described a remarkably favorable course without recurrence of pemphigus. Both developed pemphigus after COVID-19 vaccination, and their disease responded promptly to corticosteroid therapy, autoantibody titers normalized, and long-term remission was achieved. These findings stand in contrast to sporadic pemphigus, which typically follows a chronic course with frequent relapses, and they suggest that vaccine-related pemphigus may represent a distinct, transient clinical entity in some cases.

Previous reports have described pemphigus occurring after COVID-19 mRNA vaccination, although most cases required prolonged treatment (9). Compared with these reports, our two patients had an unusually favorable course with rapid improvement and long-term remission. While the temporal relationship suggests a possible trigger, causality cannot be established, and the disease course in our cases may simply reflect transient immune activation rather than persistent autoimmunity.

The absence of DIF in Case 2 represents a limitation; however, the diagnosis was well supported by the characteristic histopathological features and markedly elevated anti-desmoglein antibody titers.

Taken together, these cases demonstrate that pemphigus may rarely develop after COVID-19 vaccination, and although most cases follow a chronic course, our observations suggest that a transient, self-limited course can occasionally occur. Such cases are exceptional and should not discourage COVID-19 vaccination.

## Data Availability

The original contributions presented in the study are included in the article/supplementary material. Further inquiries can be directed to the corresponding author.
